# Supplementary material for: Interconnections between the Cation/Alkaline pH-Responsive Slt and the Ambient pH Response of PacC/Pal Pathways in Aspergillus nidulans
Source: Cells. 2024 Apr 8;13(7):651. doi: 10.3390/cells13070651 (PMC11011638; doi:10.3390/cells13070651)
Supplement: Supplementary file 1 [file cells-13-00651-s001.zip › cells-2894116-supplementary.pdf]

Supplementary Material

Supplementary Table S1: Strains used in this study

Strain	Genotype	Reference
**MAD973**	*yA2, pabaA1, argB2, pacC^{c}700*	CIB's stock collection
**MAD1132**	*wA3, sltA1, pantoB100*	CIB's stock collection
**MAD1445**	*yA2, pabaA1, pacC^{c}14900*	Hervás-Aguilar et al., (2007)
**MAD1652**	*yA2, pabaA1, pacX20, pacC900*	Bussink et al.,(2015)
**MAD1775**	*pyrG89, pyroA4, inoB2, nkuAΔ::bar, palBΔ::pyroA, pacC900*	CIB's stock collection
**MAD1929**	*pyroA4, pacX-gfp, hhoA-mrfp, riboB2*	CIB's stock collection
**MAD2352**	*wA4, pyrG89, pyroA4, inoB2, nkuAΔ::bar, palF::ha$_3$::pyrG^{Af}, pacC900*	Bussink et al.,(2015)
**MAD3367**	*pantoB100, vps23::gfp::pyrG^{Af}*	Galindo et al., (2012)
**MAD3369**	*pyroA4, palF15, vps23::gfp::pyG^{Af}*	Galindo et al., (2012)
**MAD3419**	*inoB2, pacC900, sltA60*	CIB's stock collection
**MAD3652**	*pyrG89, pabaA1, argB2, nkuAΔ::argB, sltA::ha$_3$::pyrG^{Af}, riboB2*	Mellado et al., (2016)
**MAD3693**	*pyrG89, pabaA1, argB2, nkuAΔ::argB, sltA::ha$_3$::pyrG^{Af}, riboB2, sltBΔ::riboB^{Af}*	Mellado et al., (2016)
**MAD3816**	*sltAΔ::pyrG^{Af}, pyroA4, riboB2, argB2, nkuAΔ::argB*	Mellado et al., (2016)
**MAD3919**	*pyrG89, pabaB22, nkuAΔ::argB (argB2), sltA::ha$_3$::riboB^{Af}, riboB2*	Mellado et al., (2015)
**MAD4296**	*yA2, biA1, pabaA1, pyrG89, sltAΔ::pyrG^{Af}, argB2, pacC^{C}14900*	Mellado et al., (2016)
**MAD4499**	*pabaA1, pantoB100, pyroA4::[gpdA^{mini}::palF (cDNA)::ha$_3$::ub::pyroA^{trunc}], pacC900*	CIB's stock collection
**MAD5736**	*pyrG89, pyroA4*	CIB's stock collection
**MAD6669**	*pyrG89, pabaB22, nkuAΔ::argB(argB2), riboB2, sltA::ha$_3$::riboB^{Af}*	Picazo et al., (2020)
**MAD7621**	*nkuAΔ::argB(argB2)?, sltAΔ::pyrG^{Af}, pacX20*	This work. From cross MAD3816 x MAD1652
**MAD7622**	*wA3, sltA1, pacX20*	This work. From cross MAD1132 x MAD1652
**MAD7623**	*yA2, pyrG89?, pabaA1?, pabaB22?, nkuAΔ::argB(argB2)?, sltBΔ::riboB^{Af}, sltA::ha$_3$::pyrG^{Af}, pacX20, pacC900, riboB2?*	This work. From cross MAD3693 x MAD1652
**MAD7624**	*nkuAΔ::argB(argB2)?, pacX20, sltA::ha$_3$::riboB^{Af}*	This work. From cross MAD6669 x MAD1652
**MAD7625**	*pyrG89?, pyroA4, nkuAΔ::argB(argB2)?, sltAΔ::riboB^{Af}, pacX-gfp, hhoA-mrfp, riboB2?*	This work. From cross MAD1929 x MAD3919

**MAD7626**	*pyrG89?, pyroA4, nkuAΔ::argB(argB2)?, pacX-gfp, hhoA-mrfp, riboB2*	This work. From cross MAD1929 x MAD3919
**MAD7627**	*wA4, nkuAΔ::argB(argB2)?, nkuAΔ::bar?, pacC900, palF500*	This work. From cross MAD7621 x MAD2352
**MAD7628**	*pyroA4, inoB2, nkuAΔ::argB(argB2)?, nkuAΔ::bar?, pacX20, pacC900, palF500*	This work. From cross MAD7621 x MAD2352
**MAD7629**	*wA4, pyroA4, nkuAΔ::argB(argB2)?, nkuAΔ::bar?, sltAΔ::pyrG*^{Af}*, pacX20, pacC900, palF500*	This work. From cross MAD7621 x MAD2352
**MAD7630**	*pyroA4, nkuAΔ::argB(argB2)?, nkuAΔ::bar?, sltAΔ::pyrG*^{Af}*, pacC900, palF500*	This work. From cross MAD7621 x MAD2352
**MAD7631**	*wA4, pyroA4, inoB2, nkuAΔ::argB(argB2)?, nkuAΔ::bar?, sltAΔ::pyrG*^{Af}*, pacC900*	This work. From cross MAD7621 x MAD2352
**MAD7632**	*pyroA4, pabaA1, nkuAΔ::argB(argB2)?, argB2?, pacC*^{C}*700, riboB2*	This work. From cross MAD973 x MAD3816
**MAD7633**	*yA2, pyroA4, pabaA1, nkuAΔ::argB(argB2)?, argB2?, sltAΔ::pyrG*^{Af}*, pacC*^{C}*700, riboB2*	This work
**MAD7634**	*wA4, inoB2, nkuAΔ::argB(argB2)?, nkuAΔ::bar?, sltAΔ::pyrG*^{Af}*, pacC900, pyroA4::[gpdA*^{mini}*::palF (cDNA)::ha*$_{3}$*::ub::pyroA*^{trunc}*]*	This work
**MAD7669**	*pyrG89, pyroA4, nkuAΔ::bar, vps23::gfp::pyrG*^{Af}	This work
**MAD7670**	*pyrG89, pabaB22, nkuAΔ::bar, sltAΔ::riboB*^{Af}*, riboB2, vps23::gfp::pyrG*^{Af}	This work
**MAD7671**	*wA3, pantoB100, pacC900, pacX20, sltA1*	This work. From cross MAD1132 x MAD1652
**MAD8108**	*pyrG89, nkuAΔ::bar, pyroA4,inoB2, palBΔ::pyroA, sltAΔ::pyrG*^{Af}	This work

Supplementary Table S2: Oligonucleotides used in quantitative PCR experiments

Primer	Sequence (5’-3’)
qPCR_sltA_1	TCCTCAGCAACAGACTACCTCGC
qPCR_sltA_2	CAAGATCGAACGGTTCAGACGG
qPCR_sltB_1	ATCAACCAGGATCGCTTAGACGC
qPCR_sltB_2	GTACGTTCACCATCGTCAACGC
qPCR_pacC_1	CTACATTGCCAACCGTCTTGAGC
qPCR_pacC_2	GGGATACATCACACTGTCCTCGG
qPCR_palF_1	CTCTTGCCTAGTCAACCTCCGTG
qPCR_palF_2	CGCTCCAACTCCTGTTTATCCTC
qPCR_pacX_1	TCAGCAGTAAGGGAGGTGTCTCC
qPCR_pacX_2	GATCCTGATCCCGCTCCATATC
qPCR_benA_1	AGATGCGCAACATCCAGAGC
qPCR_benA_2	CTGGTACTCGGAGACGAGATCG

Supplementary figures

Figure S1: **Gene expression profiles in wild-type and *sltAΔ* strains grown under stress caused by high extracellular sodium concentration**. A) Expression profiles of *sltA* and *sltB* genes in mycelia of wild-type strain grown in standard culture condition (Control, C) and then transferred to medium containing 1 M NaCl. Panels B and C show the expression levels of *pacC* (B) and *palF* (C) genes in mycelia from wild-type and null *sltA* strains grown in non-stressing condition (Control, C) and in the presence of 1 M NaCl. Wild-type and null *sltA* strains used in this experiment were MAD3652 and MAD3816, respectively. Error bars represent the standard deviations of three replicates for each sample result. *, P<0.01-0.05; **P<0.001-0.01; ***,P<0.0001-0.001; ****, P<0.00001. Not significant P-values (≥0.05) are not indicated in graphs.

Figure S2: **Patterns of PacC proteolysis at alkaline pH along extended experimental times and with calcium supplementation**. A) Detection of PacC forms in ambient alkaline pH along 4 hours in wild-type and *sltAΔ* strains. Strains and methodology used are the same as in Figure 2. Exploring longer experimental times aimed to detect a delayed proteolytic processing of $PacC^{72kDa}$ after medium alkalinisation in the absence of SltA activity. B) Similarly, the effect of 10 mM $CaCl_2$ was studied on the pattern of proteolytic processing of in the wild-type and null *sltA* backgrounds. Neither longer exposure to alkaline pH nor addition of calcium modified the pattern of PacC proteolysis observed in the null *sltA* mutant. C) Detection of PacC protein forms in the *sltA60* mutant background (MAD3419) at alkaline pH. The mild loss-of-function phenotype caused by *sltA60* allele allowed the detection of $PacC^{53kDa}$ form and recovered higher levels of $PacC^{27kDa}$ with a correct electrophoretic mobility although notable levels of $PacC^{72kDa}$ were observed compared to a wild-type background (see text for details).

Figure S3: **Characterization of the zinc binuclear cluster protein PacX**. A) Analysis of expression levels of PacX-GFP and degradation forms in WT and null *sltA* background at pH 8. On the right, relative quantification of protein levels of PacX and degradated PacX (PacX deg). B) *In vivo* detection of PacX-GFP protein by fluorescence microscopy. Nuclei were detected by tagging histone 1 (HhoA) with monomeric red fluorescent protein (mRFP). Cells were visualised in control condition and after 15 and 120 minutes of alkalinity induction. PacX-GFP cellular localisation was not altered in any strain or condition tested.

Figure S4: Effects of *pacX20* mutation in PacC protein levels and proteolytic processing. A) Comparative immunodetection of PacC forms in *wild-type*, *sltAΔ* (top) and *pacX20* single mutants and the double mutant *sltAΔ pacX20* (bottom). Exposure times were comparable and images highlight the positive effect of *pacX20* mutation on PacC levels. B) Lack of effect of *pacX20* mutation on the incorrect processing of PacC in *sltAΔ*, *sltBΔ* and *sltA1* mutant backgrounds.

FigureS1

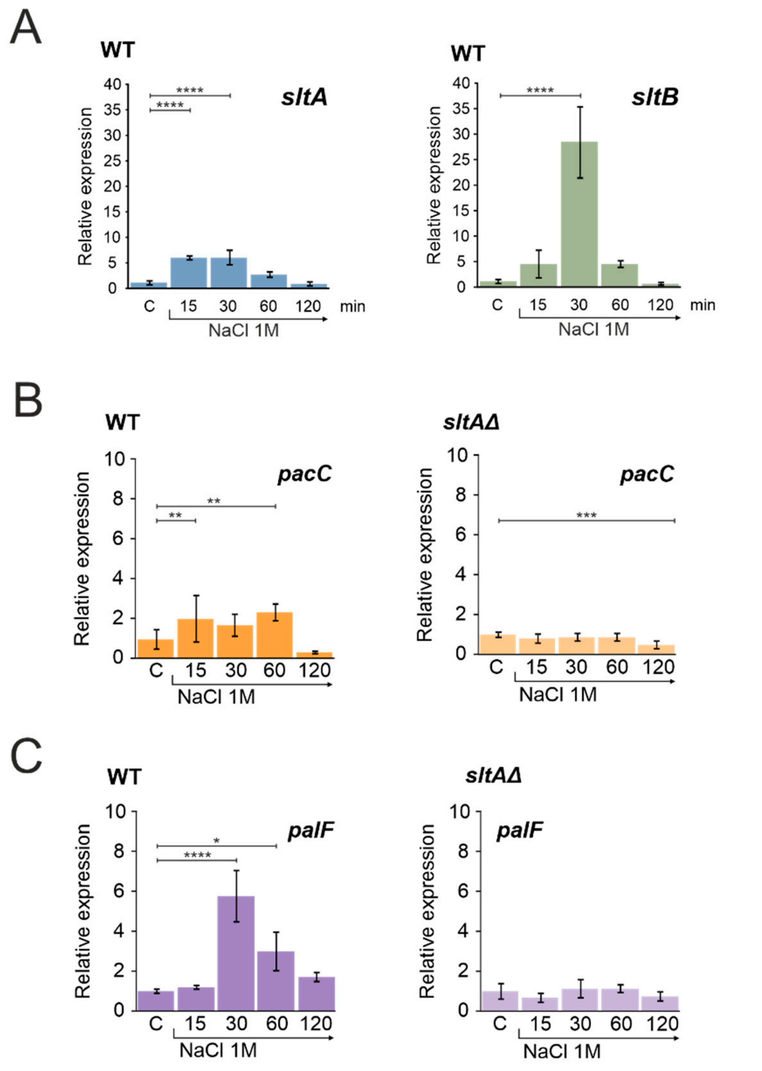

A
WT
sltA
Relative expression
C 15 30 60 120 min
NaCl 1M
WT
sltB
B
WT
pacC
sltAΔ
pacC
C
WT
palF
sltAΔ
palF

FigureS2

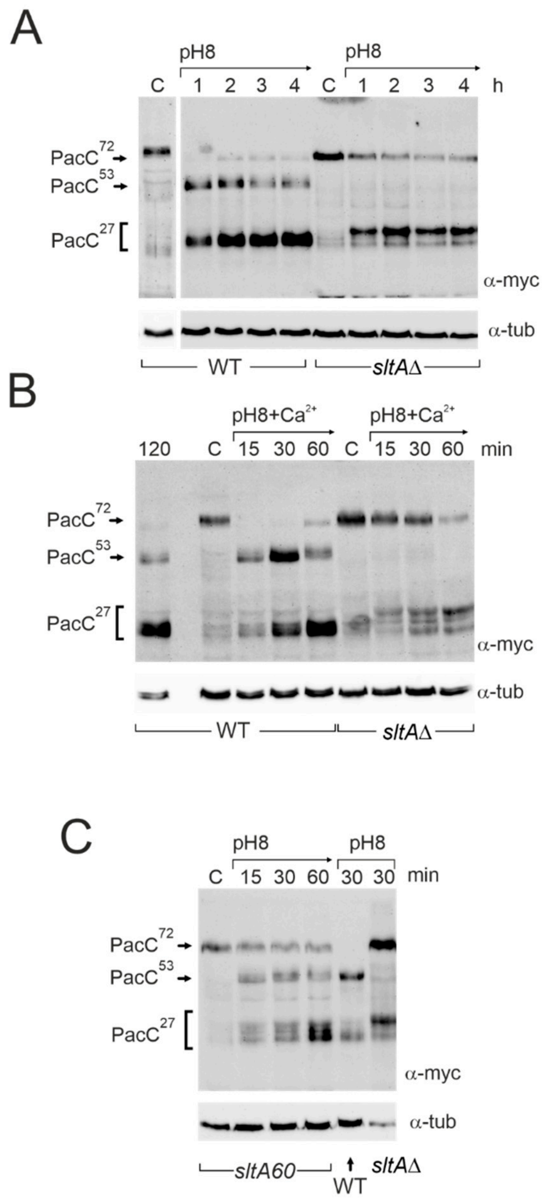

A
pH8
pH8
C 1 2 3 4 C 1 2 3 4 h
PacC72
PacC53
PacC27
α-myc
α-tub
WT
sltAΔ
B
pH8+Ca2+
pH8+Ca2+
120 C 15 30 60 C 15 30 60 min
PacC72
PacC53
PacC27
α-myc
α-tub
WT
sltAΔ
C
pH8
pH8
C 15 30 60 30 30 min
PacC72
PacC53
PacC27
α-myc
α-tub
sltA60
WT
sltAΔ

FigureS3

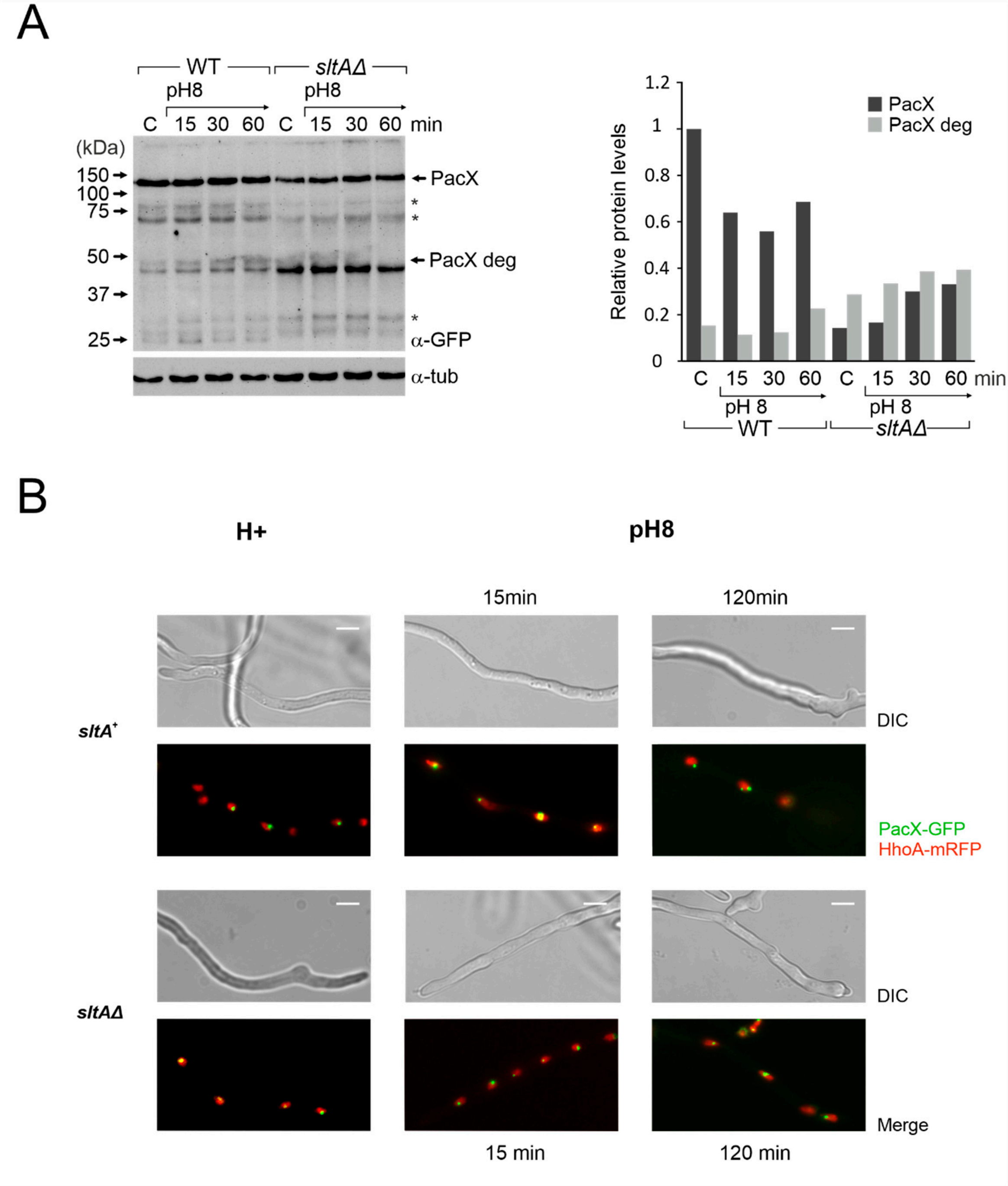
A
WT
sltAΔ
pH8
C 15 30 60 C 15 30 60 min
(kDa)
150
100
75
50
37
25
PacX
PacX deg
α-GFP
α-tub
Relative protein levels
PacX
PacX deg
pH 8
WT
sltAΔ
B
H+
pH8
15min
120min
sltA+
sltAΔ
DIC
PacX-GFP
HhoA-mRFP
Merge
15 min
120 min

FigureS4

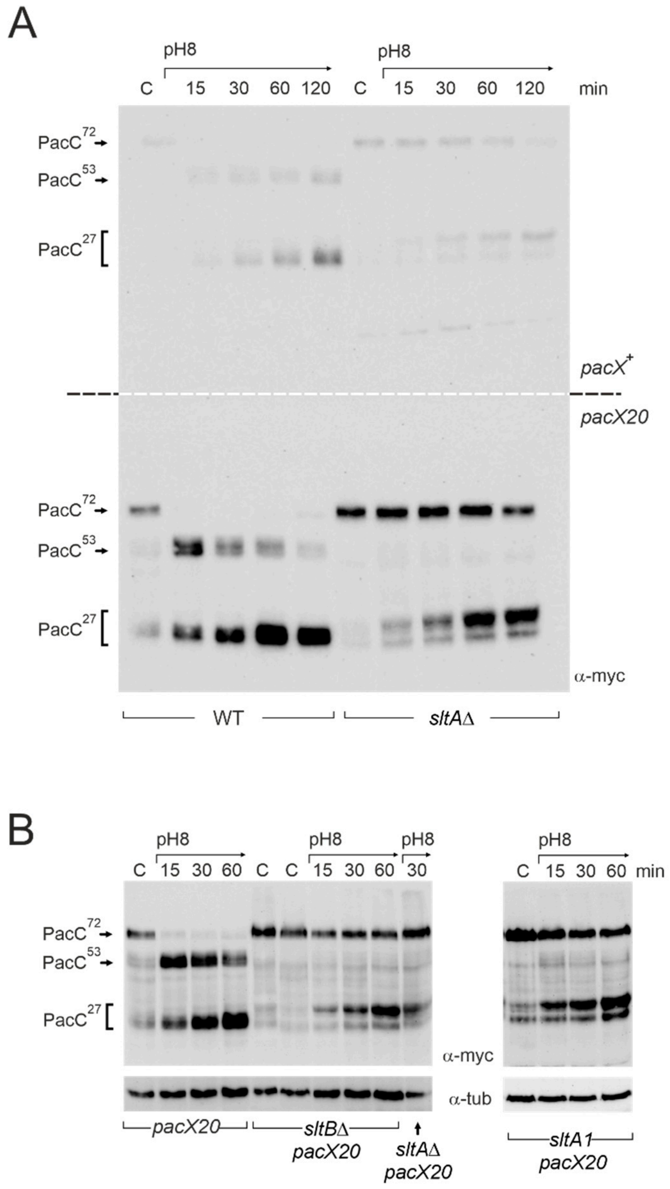

A
pH8
C
15
30
60
120
min
PacC72
PacC53
PacC27
pacX+
pacX20
α-myc
WT
sltAΔ
B
pH8
C
15
30
60
min
α-myc
α-tub
pacX20
sltBΔ
pacX20
sltAΔ
pacX20
sltA1
pacX20
